# Mosaic RAS/MAPK variants cause sporadic vascular malformations which respond to targeted therapy

**DOI:** 10.1172/JCI98589

**Published:** 2018-03-12

**Authors:** Lara Al-Olabi, Satyamaanasa Polubothu, Katherine Dowsett, Katrina A. Andrews, Paulina Stadnik, Agnel P. Joseph, Rachel Knox, Alan Pittman, Graeme Clark, William Baird, Neil Bulstrode, Mary Glover, Kristiana Gordon, Darren Hargrave, Susan M. Huson, Thomas S. Jacques, Gregory James, Hannah Kondolf, Loshan Kangesu, Kim M. Keppler-Noreuil, Amjad Khan, Marjorie J. Lindhurst, Mark Lipson, Sahar Mansour, Justine O’Hara, Caroline Mahon, Anda Mosica, Celia Moss, Aditi Murthy, Juling Ong, Victoria E. Parker, Jean-Baptiste Rivière, Julie C. Sapp, Neil J. Sebire, Rahul Shah, Branavan Sivakumar, Anna Thomas, Alex Virasami, Regula Waelchli, Zhiqiang Zeng, Leslie G. Biesecker, Alex Barnacle, Maya Topf, Robert K. Semple, E. Elizabeth Patton, Veronica A. Kinsler

**Affiliations:** 1 Genetics and Genomic Medicine, University College London (UCL) Great Ormond Street Institute of Child Health, London, United Kingdom.; 2 Paediatric Dermatology, Great Ormond Street Hospital for Children NHS Foundation Trust, London, United Kingdom.; 3 MRC Human Genetics Unit and Cancer Research UK (CRUK) Edinburgh Centre, Medical Research Council (MRC) Institute of Genetics and Molecular Medicine, University of Edinburgh, Western General Hospital, Edinburgh, United Kingdom.; 4 Metabolic Research Laboratories, Wellcome Trust–MRC Institute of Metabolic Science, University of Cambridge, Cambridge, United Kingdom.; 5 The National Institute for Health Research Cambridge Biomedical Research Centre, Cambridge, United Kingdom.; 6 Department of Biological Sciences, Birkbeck, University of London, London, United Kingdom.; 7 Molecular Neuroscience, UCL Institute of Neurology, London, United Kingdom.; 8 Department of Medical Genetics, University of Cambridge, Cambridge Biomedical Campus, Cambridge, United Kingdom.; 9 Plastic Surgery, Great Ormond Street Hospital for Children NHS Foundation Trust, London, United Kingdom.; 10 Dermatology and Lymphovascular Medicine, St. George’s Hospital NHS Trust, London, United Kingdom.; 11 Paediatric Oncology, Great Ormond Street Hospital for Children NHS Foundation Trust, London, United Kingdom.; 12 Manchester Centre for Genomic Medicine, St. Mary’s Hospital, Manchester, United Kingdom.; 13 Developmental Biology and Cancer Programme, UCL Great Ormond Street Institute of Child Health and Department of Histopathology, Great Ormond Street Hospital for Children NHS Foundation Trust, London, United Kingdom.; 14 Paediatric Neurosurgery, Great Ormond Street Hospital for Children NHS Foundation Trust, London, United Kingdom.; 15 National Human Genome Research Institute, NIH, Bethesda, Maryland, USA.; 16 Paediatrics and Clinical Genetics, Kaiser Permanente Medical Center, Sacramento, California, USA.; 17 Clinical Genetics, St. George’s Hospital NHS Trust, London, United Kingdom.; 18 Paediatric Dermatology, Birmingham Women’s and Children’s NHS Foundation Trust Birmingham and University of Birmingham, Birmingham, United Kingdom.; 19 McGill University Health Centre and Research Institute, Montréal, Quebec, Canada.; 20 Paediatric Pathology, Great Ormond Street Hospital for Children NHS Foundation Trust, London, United Kingdom.; 21 Interventional Radiology, Great Ormond Street Hospital for Children NHS Foundation Trust, London, United Kingdom.; 22 University of Edinburgh Centre for Cardiovascular Science, Queen’s Medical Research Institute, Edinburgh, United Kingdom.

**Keywords:** Therapeutics, Vascular Biology, Drug therapy, Molecular genetics, Signal transduction

## Abstract

**BACKGROUND.** Sporadic vascular malformations (VMs) are complex congenital anomalies of blood vessels that lead to stroke, life-threatening bleeds, disfigurement, overgrowth, and/or pain. Therapeutic options are severely limited, and multidisciplinary management remains challenging, particularly for high-flow arteriovenous malformations (AVM).

**METHODS.** To investigate the pathogenesis of sporadic intracranial and extracranial VMs in 160 children in which known genetic causes had been excluded, we sequenced DNA from affected tissue and optimized analysis for detection of low mutant allele frequency.

**RESULTS**. We discovered multiple mosaic-activating variants in 4 genes of the RAS/MAPK pathway, **KRAS**, **NRAS**, **BRAF**, and **MAP2K1**, a pathway commonly activated in cancer and responsible for the germline RAS-opathies. These variants were more frequent in high-flow than low-flow VMs. In vitro characterization and 2 transgenic zebrafish AVM models that recapitulated the human phenotype validated the pathogenesis of the mutant alleles. Importantly, treatment of AVM-BRAF mutant zebrafish with the BRAF inhibitor vemurafinib restored blood flow in AVM.

**CONCLUSION.** Our findings uncover a major cause of sporadic VMs of different clinical types and thereby offer the potential of personalized medical treatment by repurposing existing licensed cancer therapies.

**FUNDING.** This work was funded or supported by grants from the AVM Butterfly Charity, the Wellcome Trust (UK), the Medical Research Council (UK), the UK National Institute for Health Research, the L’Oreal-Melanoma Research Alliance, the European Research Council, and the National Human Genome Research Institute (US).

## Introduction

**Figure 1:**
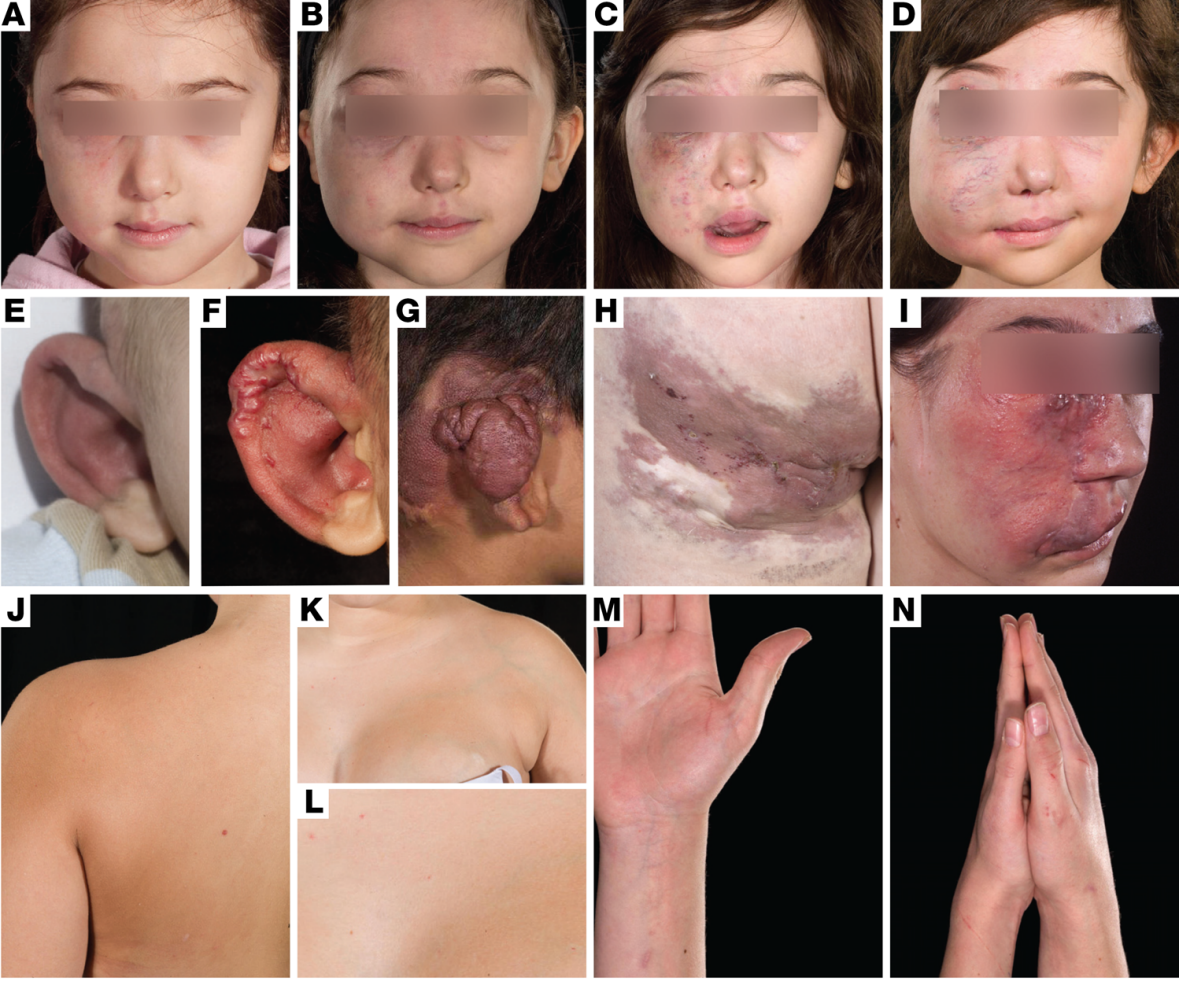
A broad clinical spectrum of VMs in somatic RAS/MAPK mutations. Inexorable enlargement of high-flow AVMs with age, affecting the face and leading to loss of vision in the right eye (**A**–**D**), and affecting the right ear helix and posterior auricular soft tissues leading to eventual resection of the helix (**E**–**G**). Varied clinical examples of the spectrum of high-flow VMs (AVMs) of the temple, left leg/buttock, and left face (**H** and **I**). Segmental overgrowth of the left arm, chest wall, and breast with a colocalized low-flow VM, detectable by a uniform brownish-pink macular capillary malformation and superimposed scattered telangiectasia, with clear midline demarcation (**J**–**L**). Segmental overgrowth of the right arm and hand, with a colocalized uniform brownish-pink capillary malformation with superimposed scattered telangiectasia (**M** and **N**).

**Figure 2:**
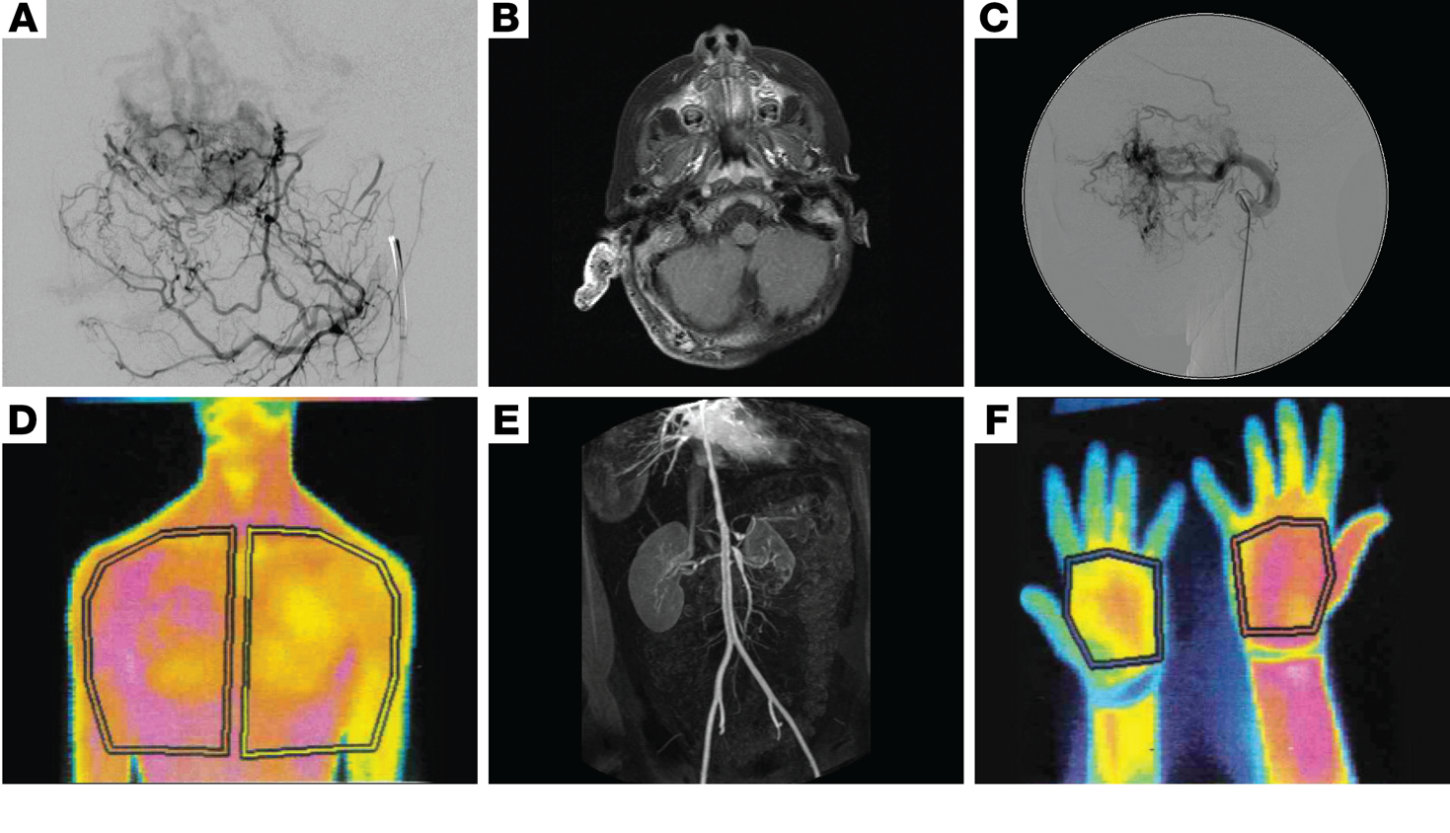
Imaging of sporadic VMs secondary to mutations in MAPK pathway genes demonstrating involvement of all blood vessel sizes. (**A**) Lateral image from a digitally subtracted angiogram showing a leash of small, abnormal vessels shunting through a dense capillary bed to early filling veins. (**B**) Axial contrast-enhanced fat-saturated T1 weighted MRI image showing a grossly enlarged right pinna and thickened posterior auricular soft tissues. The abnormal tissue is filled with multiple signal voids, representing enlarged abnormal vessels. The pinna enhances avidly. (**C**) Lateral image from a digitally subtracted angiogram, with the catheter tip in the grossly enlarged left internal maxillary artery, which supplies a leash of abnormal high-flow vessels in the face. (**D**) Thermography of low-flow VMs with overgrowth of the left chest wall and arm demonstrates increased temperature (shown in magenta) compared with the right-sided structures (**E**), and in the right forearm and thumb compared with the left (**F**). 3D reconstruction of an abdominal CT angiogram demonstrating multifocal vascular disease, with severe stenoses of the descending aorta, coeliac axis, superior mesenteric artery origin, and right renal artery (**E**).

Sporadic vascular malformations (VMs) are congenital malformations of blood vessels with high associated morbidity and limited treatment options (Figure 1 and Figure 2). VMs have traditionally been divided into diagnostic groups according to anatomical site (intracranial versus extracranial) and flow characteristics (high versus low), with detailed subclassification incorporating multiple historical diagnostic labels (1). In high-flow, or arteriovenous, malformations (AVM), direct complex interconnections between arteries and veins without the normal interposed small-bore capillary network permit dangerous flow of high-pressure arterial blood into thin-walled veins, leading to bleeds or stroke. In low-flow, capillary and/or venous malformations, interconnections between anatomically abnormal capillaries and/or veins can lead to sludging of blood, perilesional tissue anoxia, thrombosis, and overgrowth. Application of next-generation sequencing to a range of sporadic syndromes featuring VMs has rapidly established the paradigm that such disorders are commonly caused by postzygotic variants activating key cellular growth pathways (2–11). Variants overlap with those documented in cancer, but the allelic spectra may differ. Accurate genotype-based stratification of affected patients has begun to improve prognostication and, in conditions affecting the PI3K/AKT/MTOR signaling pathway, has led to early clinical trials of targeted therapies (12, 13). Despite these discoveries, however, a substantial proportion of patients have no mutations in known associated genes (9, 14, 15). We sought to address this issue using ultradeep next-generation sequencing and bioinformatic analyses aimed at detection of low mutant allele frequency in affected tissue. Here, we discover that multiple mosaic-activating variants in 4 genes of the RAS/MAPK pathway cause VM and model therapy in an animal model.

## Results

**Figure 3:**
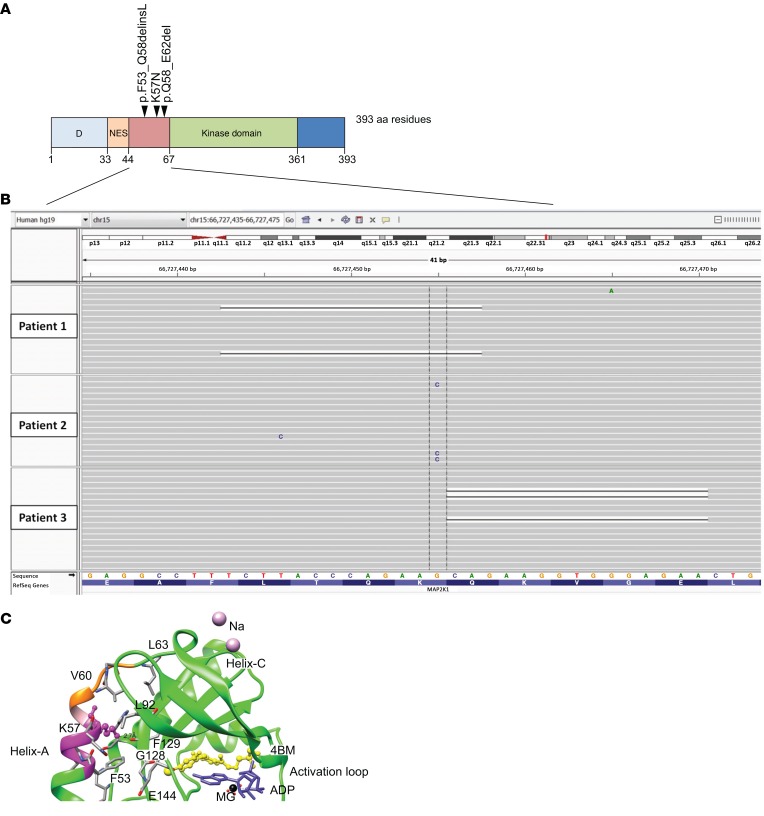
Somatic variants in *MAP2K1* cluster in exon 2 and are predicted to destabilize the 3D structure of the inactive form of the kinase. (**A**) Schematic representation of clustered somatic mutations in exon 2 of *MAP2K1*. (**B**) Low allele frequency mutations on IGV visualization of deep next-generation sequencing data from VM tissue samples of 3 patients. (**C**) 3D structural modelling of an inhibitor-bound (4BM) form (PDB ID: 3EQG) of MAP2K1 demonstrating the mutated residues. Deletions are highlighted in magenta (residues 53–58) and orange (residues 58–62, with residue 58 common to both in pink). Critical residue K57, which is substituted as a result of the missense mutation, is shown using ball and stick representation, with the dashed line indicating a hydrogen-bond interaction with the β-sheet of the kinase. Residues involved in interaction between helix A and the core kinase domain are also shown.

Twenty-five patients with high-flow and 135 patients with low-flow VMs, in whom known VM-related pathogenic variants had previously been excluded (Methods), were investigated to identify the cause of the clinical phenotype. Using deep next-generation sequencing of affected tissue, known or predicted pathogenic variants in RAS/MAPK pathway genes were identified in 9 of the 25 patients with AVMs (Tables 1 and 2 and Figure 3) and confirmed by a second method. Identified variants were in *KRAS* (*n* = 4) and *BRAF* (*n* = 1), encoding key proximal components of the RAS/MAPK signaling pathway, known to be pathogenic, but previously undescribed as causal in any type of VM. In addition, a cluster of variants was identified in exon 2 of *MAP2K1* (*n* = 4), confirming a hot spot very recently reported in extracranial AVM, but not known prior to our study (9), and adding 1 small intragenic deletion (c.159_173del, p.[F53_Q58delinsL]) (Table 2 and Figure 3) to the allelic spectrum. Variant allele frequencies ranged from 3% to 26% in affected tissue, but with no detectable variant in paired blood samples where these were available (*n* = 4/9, Table 2). One *KRAS*-variant patient had a sporadic AVM restricted to the intracranial cavity; this patient presented with an intracranial hemorrhage at the age of 13. The other 8 RAS/MAPK variant patients had AVMs centred in the skin or soft tissues at various anatomical sites, with intracranial extension documented in 2 (Table 1).

Of the 135 patients with low-flow VMs, 5 had mosaic RAS gene variants, 4 in *KRAS* and 1 in *NRAS*, known to be pathogenic. Variants were present in each affected tissue tested and were consistent within an individual, with a variant allele load of 3% to 29%, but undetectable in blood (*n* = 4/5, Table 2). These patients exhibited a spectrum of phenotypic manifestations, as is typical of mosaic disorders (Table 1).

Structural modeling was undertaken of the 4 mosaic variants detected in exon 2 of the *MAP2K1* gene, 2 identical missense variants (p.[K57N]), and 2 small intraexonic deletions removing, respectively, codons 53–58 (novel c.159_173del, p.[F53_Q58delinsL]) and 58–62 (c.173_187del, p.[Q58_E62del]) (Uniprot Q02750.2 (MP2K1_HUMAN). All were found to affect helix A in the 3D protein structure (residues 44–58). The deletions of residues 53–58 and 58–62 are predicted to affect the integrity of helix A, and K57 is a critical amino acid involved in a hydrogen bond interaction with the backbone of a β strand at the active site (Figure 3C), a critical 3D structure stabilizing interaction. Numerous interactions involving Q46, R49, L50, F53, K57, V60, and the rest of the kinase domain were found in the inhibited form of MAP2K1 (Figure 3C), consistent with critical involvement of helix A in protein function. Helix A forms an integral part of the negative regulatory region of kinase function (16), and these variants are therefore predicted to destabilize the conformation of the inactive state.

**Figure 4:**
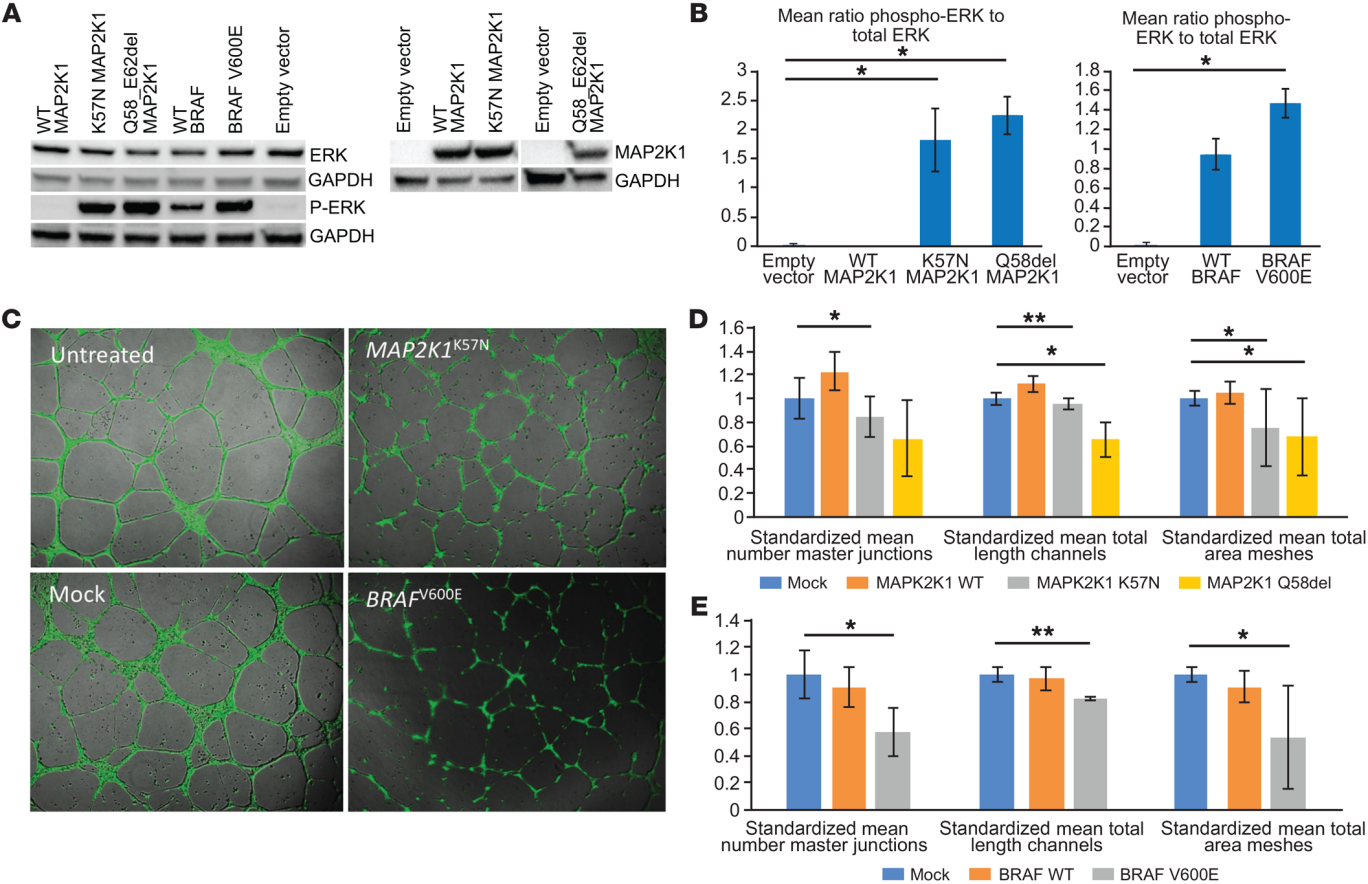
Mutations in MAPK pathway–encoding genes lead to activation of downstream signaling and disruption of vascular endothelial tube formation in vitro. (**A** and **B**) Expression of mutant BRAF^V600E^, MAP2K1^K57N^, and MAP2K1^Q58_E62del^ in HEK293T cells leads to significantly increased phosphorylation of ERK detected by immunoblotting (representative blot shown from duplicate biological replicates), compared with WT gene overexpression and controls. Data are shown as mean ± SD. *P < 0.05, 1-way ANOVA. (**C**) Expression of mutant BRAF^V600E^ and MAP2K1^K57N^ in HUVECs seeded onto Geltrex Matrix leads to visible disruption of endothelial vascular tube formation compared with controls. Original magnification, ×50. (**D** and **E**) Significant reductions in mean number of master junctions, total length of tubes, and total mesh area are indicated by asterisks. Data are shown as mean ± SD. Means were taken from triple technical replicates for each of the duplicate biological replicates, standardized to within-replicate controls, and analyzed by 1-way ANOVA with correction for multiple testing (*P < 0.0167; **P < 0.001).

Transient overexpression of *MAP2K1* and *BRAF* mutants in HEK293T cells led to increased phosphorylation of ERK compared to either overexpression of corresponding WT genes or to mock-transfected controls at 24 hours (Figure 4, A and B). Overexpression of 2 of the same mutants in HUVEC led to disruption and disordering of spontaneous vascular tube formation between 6 and 24 hours compared with mock-transfected controls (Figure 4C), with significant reductions in mean number of master junctions, total vascular tube length, and total mesh area (Figure 4, D and E).

**Figure 5:**
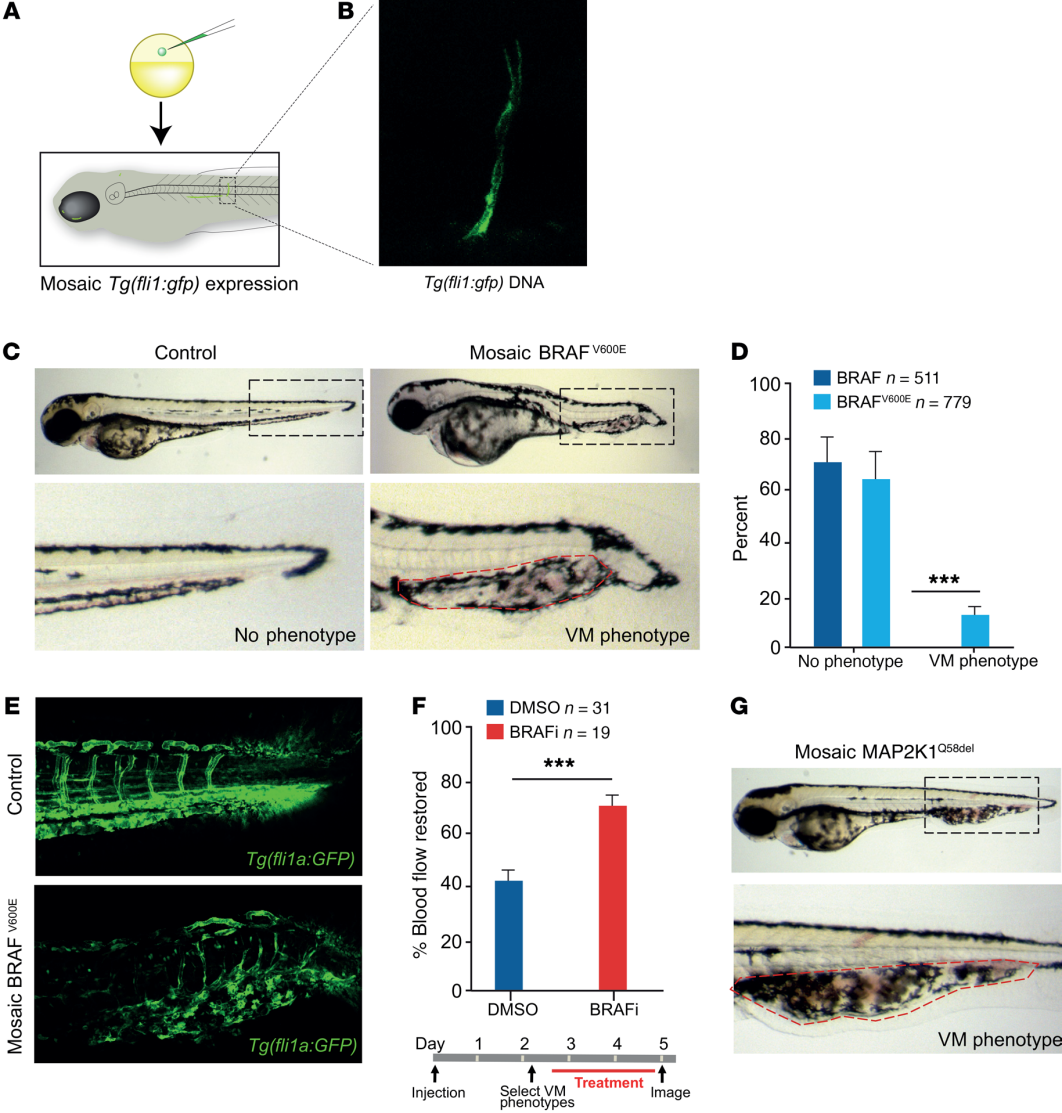
BRAF and MAP2K mutations induce VM phenotypes in zebrafish that respond to targeted therapy. (**A**) Schematic of zebrafish embryos injected with *Tg* (*fli1a:GFP*) at the 1-cell stage generate zebrafish larvae that are mosaic for the transgene integration and expression. (**B**) Image of mosaic expression of *Tg* (*fli1a:GFP*) in a vessel. (**C**) Images of zebrafish expressing WT or mutant BRAF^V600E^ in a mosaic fashion from a *fli1a* promoter in endothelial cells. Accumulation of blood is visible at the caudal vein vascular plexus in the BRAF^V600E^–expressing zebrafish and outlined with a dashed red line. (**D**) Quantification of VM phenotype in BRAF^WT^-expressing (*n* = 511) and BRAF^V600E^-expressing (*n* = 779) zebrafish. (**E**) BRAF^WT^ and BRAF^V600E^ mosaic expression in stable *Tg* (*fli1a:GFP*) larvae to visualize all vessels in the zebrafish in the VM lesion. Increased numbers of vascular channels and disorganized architecture of VM lesions are clearly detectable. (**F**) Schematic of VM treatment protocol and quantification of the percentage of VM BRAF^V600E^ zebrafish with improved blood flow following treatment with vemurafenib. VM BRAF^V600E^ zebrafish were randomized prior to DMSO (*n* = 31) or vemurafenib (*n* = 19) treatment and blind scored. (**G**) Images of zebrafish expressing MAPK2K1^Q58del^ in a mosaic fashion from a *fli1a* promoter in endothelial cells (*n* = 5/37). Zebrafish larvae in **D** were analyzed by an unpaired parametric *t* test with Welch’s correction and in **F** by a paired *t* test comparing matched pairs (before and after treatment). Data are shown as SEM. ***P < 0.001.

Two zebrafish models of VM were then developed to validate our findings in vivo and to serve as a platform for screening of potential drug treatments. Artery and vein development in zebrafish are regulated by mechanisms similar to those in humans (17), and blood flow through the vasculature is clearly visible by 2 days of development (Supplemental Video 1; supplemental material available online with this article; https://doi.org/10.1172/JCI98589DS1). We injected separately BRAF^V600E^ and MAPK2K1^Q58del^ expressed from a pan-endothelial promoter, *fli1a*, into single-cell zebrafish embryos to generate postzygotic expression of BRAF^V600E^ or MAPK2K1^Q58del^ in vessels that, as in the patients, is expressed in a mosaic fashion (Figure 5, A and B). For some animals, both BRAF and BRAF^V600E^ expression led to shortening along the anterior-posterior axis, as reported previously in zebrafish with high MAPK expression, and these were removed from the analysis (Supplemental Figure 1) (18, 19). Notably, BRAF^V600E^, but not expression of WT BRAF, led to disordered vessel formation, leading to severely impeded blood flow, and recapitulated the clinical features of patient VMs (Figure 5, C–E, and Supplemental Video 1). Interestingly, these VMs formed preferentially in the caudal vein vascular plexus (20). Mosaic expression of MAP2K1^Q58del^, but not WT MAP2K1, also generated VM (*n* = 5/37) (Figure 5G). Zebrafish with established VM were then treated for 2 days with a low, continuous dose of vemurafenib (0.1 μM), the approved anticancer BRAF inhibitor, and assessed by blind scoring for improved blood flow (Figure 5F). Strikingly, almost all vemurafenib-treated zebrafish exhibited improved blood flow (Figure 5F and Supplemental Video 1). In contrast, drug treatment had no effect on control zebrafish, and only a minority of BRAF^V600E^ VMs demonstrated improved blood flow spontaneously. These important results of improvement in phenotype from RAS-RAF-MAPK–targeted therapy in an animal model echo recent murine models of VM ameliorated by targeted pathway inhibition of the PI3K/AKT/MTOR pathway (21, 22).

## Discussion

We discovered mosaic-activating variants in oncogenes at several levels of the RAS/MAPK signaling pathway as a major cause of sporadic VMs and particularly of the clinically high-risk group of AVMs. This discovery identifies the RAS/RAF/MAPK pathway as a major cause of sporadic VMs and opens the door to repurposing of targeted medical therapies on a personalized medicine basis. The pathogenicity of these variants is supported by several lines of evidence, including the well-established pathogenicity of the *BRAF*, *KRAS*, and *NRAS* variants in other disorders and tissues, the mosaic occurrence of these variants in the lesions and their absence in peripheral blood, and the existence of a hotspot of *MAP2K1* variants in the Catalogue Of Somatic Mutations In Cancer (COSMIC) database (http://cancer.sanger.ac.uk/cosmic), comprising 65 tumor variants from F53 to E62.

**Figure 6:**
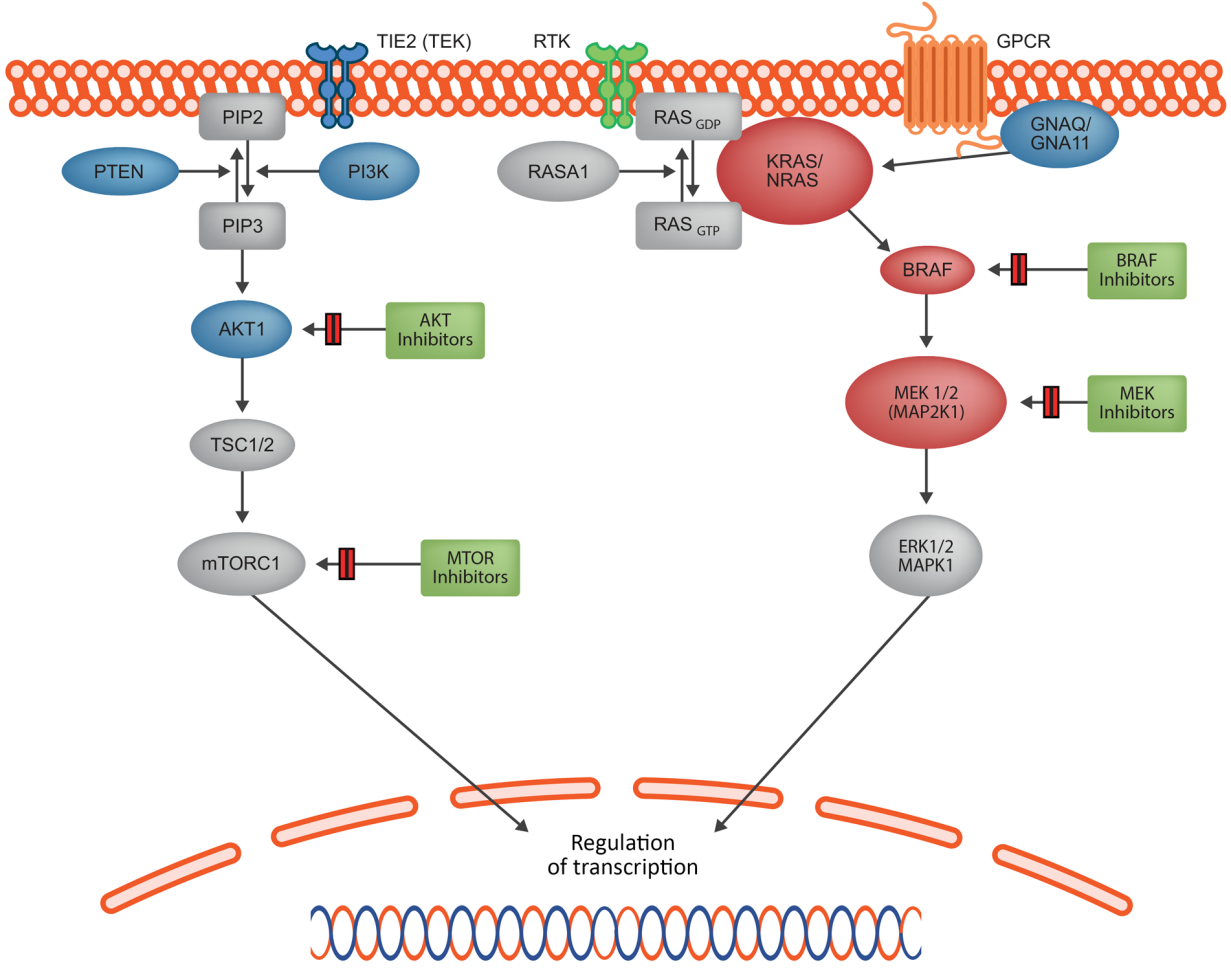
Schematic summary of the known and newly identified signaling proteins affected by mosaic mutations that lead to a VM phenotype. Key signaling pathways PI3K/AKT/MTOR and RAS/RAF/MEK/ERK control cellular growth, apoptosis, and differentiation through complex transcriptional regulation. Multiple receptor types feed into one or both pathways. In addition, there is crosstalk between the 2 pathways at multiple levels (not shown). Proteins affected by the genetic mutations presented in this paper are shown in red, with previously identified sites shown in blue. Key classes of potential targeted therapeutics are shown in green boxes. RTK, receptor tyrosine kinase; GPCR, G protein–coupled receptor.

Several genetic causes of human inherited and sporadic VM are already known (23). Inherited susceptibility to cerebral cavernous (low-flow) malformations is conferred by germline mutations in *KRIT1* (24), *CCM2* (25), and *PDCD10* (26); however, malformations themselves require a somatic “second hit” (27). The same paradigm of an inherited genetic diathesis interacting with a somatic mutation applies to heritable extracranial low-flow malformations caused by mutations in *TEK* (10) and *GLMN* (28, 29) and heritable (high-flow) AVMs associated with mutations in *PTEN*, *ACVR1*, *ENG*, *RASA1* (30, 31). The same mechanism is also likely in the recently described gene for CM-AVM syndrome *EPHB4* (32). Known causes of sporadic VMs are postzygotic mutations in *TEK* (10, 11), *AKT1* (2), *PIK3CA* (7), *GNAQ* (3, 4), *GNA11* (4), and *MAP3K3* (8) in low-flow VMs and recently described *MAP2K1* mutations in high-flow extracranial malformations (Figure 6). Thus, there is a pattern of milder predisposing mutations in the germline requiring a second hit to lead to phenotype and more severe mutations leading directly to a phenotype only seen as postzygotic hits. Our discovery that RAS/MAPK VM mutations are strong activators of the MAPK signaling pathway and overlap with the cancer allele spectrum is in line with the hypothesis that lethal genes survive by mosaicism (33).

In silico protein-interaction analysis (34) of the full spectrum of genes associated with VMs, including our new findings, demonstrates a strong network of functional interaction among the gene products (Supplemental Figure 2). This aligns with recent mechanistic work on VMs secondary to germline variants in *CCM2*, *PCD10*, and *KRIT1*, which drive VMs by increasing MAP3K3 (MEKK3) signaling in endothelial cells (35). MAP3K3 is essential for the development of embryonic cardiovascular systems in mice (36) and zebrafish (37) and is considered a critical nexus between the congenital cavernous malformation complex and downstream signaling pathways (35, 37, 38), such as those mediated by Rho signaling (38), p38 MAPK (39), and MEK5/ERK5 (37, 39). We hypothesize that congenital VMs ultimately result from dysregulation of vascular cell MAPK and/or PI3K signaling during human embryonic development.

Analysis of multiple different affected tissues in 2 of the patients revealed consistency of mutation within an individual, confirming a postzygotic hit to a multipotent precursor in those cases. Although several mosaic disorders have been described in association with postzygotic activating variants in *RAS* oncogenes, VMs have not been described in those phenotypes (40–46). Arterial abnormalities have been described in association with epidermal nevi, but without genotype data (47–49). Interestingly, postnatal somatic second-hit RAS and RAF gene family variants have been described in the small acquired vascular tumors pyogenic granulomas, arising on a background of a postzygotic *GNAQ*-variant congenital capillary malformation (50). In these cases, RAS variants were not the cause of the underlying VM, but consistent with somatic RAS variants leading to tumors of many types.

These findings are particularly important in the context of the lack of effective therapy for complex VMs, particularly for AVMs. Embolization and surgical intervention are not always possible for safety reasons and, where possible, can have limited short-term results due to very frequent localized recanalization and recurrence or be associated with disfiguring results. Identification of mosaic mutations in a third of our AVM cohort, including intracranial AVMs, will radically alter the concept of management of these VMs by introducing the possibility of targeted medical therapy.

The cause of the difference in frequency of these variants between the high- and low-flow cohorts is not clear. As a minority of the low-flow cohort skin biopsies were cultured for fibroblasts before DNA extraction, it is possible that the mutations were either lost in culture or not present in fibroblasts. We would not, however, expect this to explain the size of the difference between the groups, and further work will be needed to explore the increased frequency in AVMs.

In summary, these findings demonstrate a cause of sporadic VMs and suggest a common pathogenesis for sporadic VMs of different vessel types and at different anatomical locations, including intracranial. Genotyping of affected tissue, where accessible, should therefore be a key element of management across the diverse medical subspecialties to which affected patients present. Resulting genetic stratification may not only be of value prognostically, but may also now serve to guide therapy. Although formal clinical trials will be required before routine repurposing of such agents is sanctioned in clinical care, licensed targeted medical therapy on a compassionate basis may already be considered for severe or life-threatening cases.

## Methods

### Patient cohorts

Twenty-three patients with AVM, confirmed clinically and radiologically, who were seen in the Paediatric Dermatology Department at Great Ormond Street Hospital for Children, were recruited. At the time of recruitment, no genes were known to be causative in sporadic AVM. After initial results of our study were available, 2 patients with intracranial-only AVM were also recruited for testing via the Paediatric Neurosurgery Department.

In addition, 135 patients with low-flow VMs and/or overgrowth, and 1 patient with a high-flow VM were recruited to the Investigation of Segmental Overgrowth Disorders study (REC 12-EE-0405) and found to be WT for a genotype of known sporadic vascular and overgrowth-related genes *PIK3CA*, *AKT1*, *GNAQ*, *GNA11*, and *TEK*. One further patient with a low-flow VM and overgrowth was identified after genotyping results from the phase 1 dose-finding trial of ARQ 092 in children and adults with Proteus syndrome study at the NIH (Bethesda, Maryland, USA), bringing the totals of high-flow to 25 and low-flow to 135. Detailed clinical phenotyping was undertaken in all patients before genotyping (Tables 1 and 2).

### DNA extraction

DNA was extracted directly from samples by the DNeasy Blood and Tissue Kit (QIAGEN) and from paraffin-embedded tissue using the RecoverAll Total Nucleic Acid Extraction Kit for FFPE (Thermo Fisher Scientific).

### Next-generation sequencing and variant calling

#### High-flow VM patients.

Twenty-one formalin-fixed paraffin-embedded (FFPE) and 4 fresh samples were sequenced using the SureSeq Solid Tumour Panel (Oxford Gene Technology). This was chosen due to the difficulty in biopsying AVMs due to risk of bleeding and because this panel is optimized for FFPE tissue. DNA library preparation was performed for each sample using the Agilent SureSelect XT Reagent Kit per the manufacturer’s protocol (http://www.ogt.co.uk/assets/0000/4457/990162_HB_SureSeqSolidTumour_110215.pdf). Samples were then pooled and sequenced on a MiSeq instrument (Illumina) according to the manufacturer’s recommendations for paired-end 150-bp reads. In-depth sequencing was performed to achieve a mean sequencing depth of 500 reads for all targeted coding bases.

For 2 further patients, fresh 4 mm skin biopsies were taken from the AVM, and DNA was extracted directly from the whole sample and from paired blood samples. These were sequenced by whole-exome sequencing. Library preparation was with SureSelect Agilent QXT v6 following the manufacturer’s protocol, and sequencing was on the HiSeq 3000 (Illumina), with a mean read depth of ×500.

Sequence alignment to the human reference genome (UCSC hg19) and variant calling and annotation were performed with our in-house pipeline. Briefly, this involves alignment with NovoAlign and removal of PCR duplicates with Picard Tools followed by local realignment around indels and germline variant calling with HaplotypeCaller according to the Genome Analysis Toolkit (GATK) best practices. We identified potentially mosaic variants with GATK muTECT2 in tumor-only somatic variant calling mode. The raw list of single nucleotide variants (SNVs) and indels was then filtered using ANNOVAR (http://annovar.openbioinformatics.org). Only exonic and donor/acceptor splicing variants were considered. Priority was given to rare variants (<1% in public databases, including 1000 Genomes project [http://www.internationalgenome.org/], NHLBI Exome Variant Server [http://evs.gs.washington.edu/EVS/], Complete Genomics 69 [http://www.completegenomics.com/public-data/69-genomes/], and Exome Aggregation Consortium [http://exac.broadinstitute.org/]). Furthermore, we have an in-house set of approximately 6,000 exomes encompassing controls, rare diseases for cross-checking any shortlisted candidate variants, and for removing sequence artefacts. Identification of candidate variants where paired samples were available was also performed using Ingenuity Variant Analysis (QIAGEN) by selecting variants present in skin but not in blood (or in mosaic levels in both). For all candidate variants, BAM files were viewed using the Integrative Genomics Viewer (Broad Institute), and mosaicism percentage was taken from the mutant allele reads divided by the total directly from the BAM. Candidate postzygotic variants were confirmed by Sanger sequencing in all DNA samples available from each patient. To maximize detection of mutant alleles at low percentage mosaicism, restriction enzyme digests of the normal allele were designed where necessary using validated methods (51) and Sanger sequencing performed. See Supplemental Table 1 for primer sequences. Touchdown PCR programs were used throughout, with 40 cycles for the first PCR (annealing and extension times of 1 minute) and 15 for the second heminested PCR where required.

#### Low-flow VM patients.

A 4 mm punch biopsy of affected tissue was taken from an area of overgrowth and/or VMs. DNA was extracted either directly from the biopsy (51/134 patients) or from dermal fibroblasts grown from the biopsy (59/134 patients), using the QiaAMP DNA Micro Kit (QIAGEN). In 9 of 134 patients, DNA was extracted from FFPE tissue samples using the QIAamp DNA FFPE Tissue Kit. In cases of facial involvement (15 of 134 patients), a buccal swab served as affected tissue, and DNA was extracted via standard methods of phenol-chloroform extraction followed by ethanol precipitation. Blood samples were collected where possible, and lymphocyte DNA was extracted via the Illustra BACC3 DNA Extraction Kit (GE Healthcare).

Targeted next-generation sequencing was performed on affected tissue DNA using a custom panel of overgrowth-related genes on an Illumina MiSeq platform. This panel was designed for the low-flow study to create a selection of possible candidate genes for overgrowth on the basis of the genes/pathways already known to be involved in this phenotype. DNA (10 ng) was amplified for 18 cycles of PCR with the Ion AmpliSeq custom DNA panel (Thermo Fisher Scientific), enriching the 195 target amplicons. This included full coverage of all coding regions of *PIK3CA*, *PTEN*, and *CCND2* and hotspot regions in 57 other genes (regions of coverage listed in Supplemental Table 2). The panel was split into 2 primer pools and amplified with 5× Ion AmpliSeq HiFi Master Mix, followed by FuPa treatment (Ion AmpliSeq DNA Library Kit 2.0; Thermo Fisher Scientific). The 2 primer pools for each sample were then pooled and purified with 1.8× Agencourt AMPure XP magnetic beads (Beckman Coulter). Amplicons were 3′ adenylated using the NebNext Ultra II End Repair/dA Tailing Module (NEB), followed by the NextFlex DNA Barcode adapter (Bio Scientific) ligation using the NebNext Ultra II ligation module (NEB). Ligation products were purified and size selected using 0.8× Agencourt AMPure XP beads. Library concentration was determined with Kapa Biosystems Library qPCR quantification kit on the Lightcycler 480 Real-Time PCR System (Roche). Libraries were subsequently diluted to 2 nM and pooled in equimolar amounts. Pooled libraries were spiked with 1% PhiX DNA (Illumina) and sequenced on the MiSeq desktop sequencer using version 2 chemistry at 250 bp read length paired end.

VCF files tailored for mosaic variant calling were created in MiSeq Reporter (Illumina) and annotated in Illumina Variant Studio, version 2.2, resulting in a list of 300–1,000 variants per sample. The programming language R was used to apply hard filters according to the following parameters: read depth greater than 5; quality score greater than 10; absence of strand bias (as determined by MiSeq Reporter), cross-sample subtraction of artefactual variants called in more than 8 samples per batch of 24; exonic nonsynonymous variants only. This resulted in a list of 1 to 4 candidate variants, the clinical relevance and sequencing quality of which were then assessed.

Mosaic variants considered to be causative were confirmed and tested in other tissue samples from the same patient alongside DNA from healthy controls, either by Sanger sequencing (primers listed in Supplemental Table 1) or by custom restriction fragment length polymorphism (RFLP). For RFLP, genomic DNA was amplified with GoTaq Green (Promega) using the primers listed in Supplemental Table 3. The PCR products were designed to include a restriction enzyme recognition site allowing specific digestion of the mutant allele, while leaving the WT allele intact. The digested PCR fragments were then mixed with GeneScan 500 LIZ Size Standard (Applied Biosystems) and loaded on an ABI3730 capillary sequencer. The area under the curve of undigested/digested DNA was used to calculate the mutation burden using GeneMapper v5.0 software (Applied Biosystems).

### Mutant plasmid construction and HEK293T and HUVEC transfection

#### pCMV6-MAP2K1.

The pCMV6-*MAP2K1* plasmid for in vivo expression in mammalian cells was ordered from Origene (ID: SC118424). To generate mutant MAP2K1 expression vector, an improved QuikChange site-directed mutagenesis protocol was used (Agilent Technologies) (52). p.(K57N) is a predicted missense alteration caused by a single nucleotide change (g>c); p.(Q58_E62del) (hereafter termed Q58del) is a deletion mutation caused by a deletion of 15 bp. The sequences of mutagenesis PCR primers were as follows: K57N-forward: 5′-GAGGCCTTTCTTACCCAGAACCAGAAGGTGGG-3′ K57N-reverse: 5′-CCCACCTTCTGGTTCTGGGTAAGAAAGGCCTC-3′ Q58del-forward: 5′-CTTACCCAGAAGCTGAAGGATGACGACTTTGAGAAGATCAG-3′ Q58del-reverse: 5′-GTCGTCATCCTTCAGCTTCTGGGTAAGAAAGGCCTCAAGG-3′. The mutagenesis PCR ran for 15 cycles, and each cycle consisted of denaturation at 95°C for 30 seconds, annealing at 58°C for 1 minute, and extension at 72°C for 5 minutes.

#### pDEST26-BRAF.

The WT and mutant (p.V600E) human BRAF cDNA are in the same Gateway middle entry clones used in a previous study (18). These 2 BRAF cDNAs were cloned into the destination vector pDEST26 for expression in mammalian cells using the Gateway Cloning System (Invitrogen) according to the manufacturer’s instructions.

#### HEK293T cell line transfection.

HEK293T cells (ATCC, catalog CRL-11268) were maintained per established protocols and were transfected with mutant and WT cDNA expression plasmids and an empty vector control using Lipofectamine 2000. One day before transfection, 6 × 10^5^ cells were plated per well of a 6-well culture vessel in 500 μl of growth medium without antibiotics so that cells would be 70%–90% confluent at the time of transfection. For each transfection sample, 4.0 μg plasmid DNA was diluted in 250 μl Opti-MEM I Reduced Serum Medium (Thermo Fisher Scientific) and mixed gently. Lipofectamine 2000 was gently mixed before use; then 10 μl was diluted in 250 μl of Opti-MEM I Medium and incubated for 5 minutes at room temperature before being combined with the diluted DNA (total volume = 500 μl), mixed gently, and incubated for 20 minutes at room temperature. Complexes (500 μl) were added to each well containing cells and medium and mixed gently by rocking the plate back and forth. Finally, cells were incubated at 37°C in a CO_2_ incubator for 30 hours prior to testing for transgene expression.

#### HUVEC transfection.

Based on transfection optimization, HUVECs (Thermo Fisher, catalog number C0035C) were incubated in transfection reagents with a 1:3 DNA/Lipofectamine LTX ratio at 37°C, 5% (v/v) CO_2_ for 48 hours. Volumes described in this section are representative of a single well in a 6-well plate. 2.5 μg of either plasmids containing the WT or mutated gene of interest (BRAF^WT^, BRAF^V600E^, MAP2K1^WT^, MAP2K1^K57N^, and MAP2K1^Q58_E62del^ as detailed above) or pDest26 empty vector or a mock transfection that represented an additional control was diluted in 500 μl in Opti-MEM I Reduced Serum Medium without serum (catalog 31985062). Next, 2.5 μl of PLUS Reagent (catalog 11514015, Thermo Fisher Scientific) was added to the DNA dilution and incubated for 10 minutes at room temperature. Transfection complexes were formed after adding 7.5 μl Lipofectamine LTX to DNA:PLUS Reagent solution (Thermo Fisher Scientific) prior to 30 minutes of incubation at room temperature and then added to a well containing 2 ml fresh growth-supplemented medium.

### Quantitative real-time PCR

To assess the efficiency of transfection of plasmid DNA in the cells, quantitative expression analysis of the genes of interest *MAP2K1* and *BRAF* as well as the endogenous control *GAPDH* was determined by real-time qPCR with TaqMan gene expression assays using the StepOnePlus (Thermo Fisher Scientific) instrument. Standard protocol per the manufacturer’s guidelines was followed for Applied Biosystems TaqMan Gene Expression Assay on the StepOnePlus instrument. Probes used for quantitative analysis were as follows: *MAP2K1* (assay ID: Hs00983247_g1), *BRAF* (assay ID: Hs00269944_m1), and *GAPDH* (assay ID: Hs02786624_g1) (all from Life Technologies). Results of the quantitative gene expression levels were obtained after the amplification reaction using StepOne software (version 2.3). Results are based on analysis of 2 biological replicates, with triplicate technical replicates in each experiment, and quantitative values of the Ct averaged. The relative expression of the genes of interest was determined by calculating the ratio of their expression compared with that of the *GAPDH* endogenous control in the same sample.

### Western blotting

Cell lysates were prepared using standard protocols. Primary and secondary antibodies are shown in Supplemental Table 4. The Western blot basic protocol was as follows: following heating at 95°C in 2× Laemmli sample buffer in 5% β-mercaptoethanol, 40 μg of protein was run on 4%–20% Mini-PROTEAN TGX Precast Protein Gels (catalog 4561096, Bio-Rad). The transfer was blocked at room temperature for 1 hour in 5% dry milk or 5% BSA dissolved in TBS–0.05% Tween 20 (TBST). The transfer was incubated with primary antibody (1:1,000 dilution in 3% BSA, BSA in 1× TBST) at room temperature overnight. The membrane was then washed 3 times for 15 minutes in TBST. Incubation with the secondary antibody was conducted at room temperature for 1 hour. As a secondary antibody, we used anti-rabbit RB96 in 1:7,000 dilution and diluted in 3% BSA (BSA in 1× TBST) or 3% milk.

### Endothelial cell tube formation assay and microscopy

Cells and all reagents in this subsection were obtained from Invitrogen unless otherwise stated. The formation of endothelial tubes by HUVECs (Thermo Fisher, catalog C0035C) on growth factor–reduced Geltrex (catalog A1413202) was conducted according to the manufacturer’s protocol (catalog MAN0001687) using Medium 200PRF supplemented with Low Serum Growth Supplement (Thermo Fisher Scientific). Briefly, 24-well culture plates were coated with 100 μl/well (50 μl/cm^2^) Geltrex and incubated for 30 minutes at 37°C. After harvesting transfected and untreated HUVECs (P2) cultured on 6-well plates, cells were seeded on coated plates at a density of 4.5 × 10^4^/cm^2^ (9 × 10^4^ cells/well) in 200 μl/cm^2^ (400 μl/well) supplemented with 200PRF medium and cultured in a CO_2_ incubator (37°C, 5% [v/v] CO_2_, 95% humidity). Thirty minutes before the end of the incubation period, cells were treated with 2 μg/ml (0.8 μl/well) calcein AM (catalog C3099) and incubated at 37°C, 5% (v/v) CO_2_. Tube formation observed at the 14-hour time point was imaged with a 5× objective lens of an Olympus IX71 inverted fluorescence and bright field microscope using HCImage software. The degree of tube formation was assessed by measuring all aspects of tubule, node, and mesh growth in triplicate, using randomly chosen fields from each well using the angiogenesis analyzer for ImageJ (NIH).

### Transgenic zebrafish

A zebrafish *fli1a* promoter was generated by gateway PCR with primers (forward: 5′-GGGGACAACTTTGTATAGAAAAGTTGCCTGGCTGTCAAGCTCCAGC-3′, reverse: GGGGACTGCTTTTTTGTACAAACTTGATATGTGGCGGAGAGACAGAG-3′; promoter-specific sequences are underlined). The promoter comprises 2.2 kb immediately upstream of the first ATG of the *fli1a* gene. The PCR product was then cloned into the gateway 5′ donor vector pDONRP4-P1R to obtain the 5′ entry clone p5Efli1a2.2k. Middle entry clones containing human BRAF^WT^, BRAF^V600E^, MAP2K1^WT^, or MAP2K1^Q58del^ cDNA were recombined with the *fli1a* promoter in p5Efli1a2.2k and the pDestTol2CG2 expression vector using the Tol2kit Gateway cloning method (53), resulting in fli1a-BRAF^WT^, fli1a-BRAF^V600E^, fli1a-MAP2K1^WT^, and fli1a-MAP2K1^Q58del^ constructs. Mixed fli1a-BRAF (1 nl) or fli1a-MAP2K1 plasmid DNA and Tol2 mRNA (37 ng/μl and 35 ng/μl, respectively) were injected into the 1-cell stage of *fli1a:GFP* zebrafish embryos (Species Danio rerio, AB line) (54). Embryos were raised at 28.5°C and screened for phenotypes. Embryos were then fixed in 4% PFA.

### Drug treatments

Zebrafish embryos were treated with the BRAF inhibitor vemurafenib (PLX4032). Embryos were incubated with 0.1 μM of the drug from 2.5 days post fertilization (dpf) after initial imaging, which was refreshed daily. Control embryos were incubated in E3 with DMSO at the same concentration as drug used. During live imaging, embryos were kept in 1:5,000 MS222 and 1.5% LMP agar. Leica stereo brightfield microscopy was used for live color imaging. Fluorescence images were taken using Leica Sp5 confocal microscopy. Images were processed using FIJI (ImageJ). GraphPad Prism was used to analyze data.

### Statistics

Western blot data (Figure 4, A and B) were analyzed using 1-way ANOVA comparing mock transfection to WT and mutant allele groups for each mutant tested. Densitometry data were pooled from biological replicates and used to calculate means and SDs. *P* < 0.05 was used at a 95% CI. Angiogenesis data (Figure 4, D and E) were analyzed using 1-way ANOVA comparing mock transfection controls to WT and mutant allele groups for each mutant tested. An initial 1-way ANOVA was undertaken to demonstrate that biological replicates were significantly different, and therefore data between replicates were first standardized to the within-experiment control before being pooled for analysis of standardized means and SD. Correction for multiple testing was applied after ANOVA, reducing the *P* value to less than 0.0167 at a CI of 95%. For zebrafish data, significance was assessed for Figure 5D using unpaired parametric *t* test with Welch’s correction and for Figure 5F using paired *t* test.

### Study approval

These studies were conducted according to Declaration of Helsinki principles and were approved by the local Research Ethics Committees of each center involved (London Bloomsbury, London, United Kingdom; University of Cambridge, Cambridge, United Kingdom; and University of Edinburgh, Edinburgh, United Kingdom). All participants provided written informed consent. Separate written informed consent was obtained for publication of all clinical photographs. All zebrafish work was done in accordance with United Kingdom Home Office Animals (Scientific Procedures) Act (1986) and approved by the University of Edinburgh Ethical Review Committee.

## Author contributions

LAO and SP were responsible for patient sample DNA extraction, sequencing and confirmation, patient recruitment and phenotyping of the low- and high-flow cohorts, HEK293T cell work, and figure preparation. PS was responsible for the HUVEC work and figure preparation. KAA and VEP were responsible for patient recruitment and phenotyping of the low-flow cohort. RK, GC, and WB contributed to sequencing and cell culture maintenance. KD, ZZ, and EEP were responsible for the zebrafish work and figure preparation. APJ and MT were responsible for the protein modeling work and figure preparation. AP and JBR contributed to the design of the sequencing data analysis. WB, NB, MG, KG, DH, SMH, TSJ, GJ, HK, LK, KMKN, AK, MJL, ML, SM, JO, CM, AM, CM, AM, JO, VEP, JBR, JCS, NJS, RS, BS, AT, AV, and RW contributed patient samples and phenotypic data to the cohort, and HK, KMKN, MJL, and JCS also contributed to the mutation discovery of their patient. LGB contributed patient genotype/phenotype and critical review of the manuscript. AB reviewed all the imaging and contributed radiological images for figures. RKS conceived, designed, and directed the research for the low-flow cohort as well as recruiting a large number of patients and the genotypic and phenotypic data to that cohort, in addition to contributing to and providing critical reviews of the manuscript. EEP conceived, designed, and directed the zebrafish research and contributed to and provided critical review of the manuscript. VAK conceived and designed the study, directed the research, and recruited patients to the high-flow cohort, recruited patients to the low-flow cohort, analyzed the sequencing and angiogenesis data, wrote the manuscript, and prepared figures.

## Supplementary Material

Supplemental data

Supplemental Video 1

## Figures and Tables

**Table 1:**
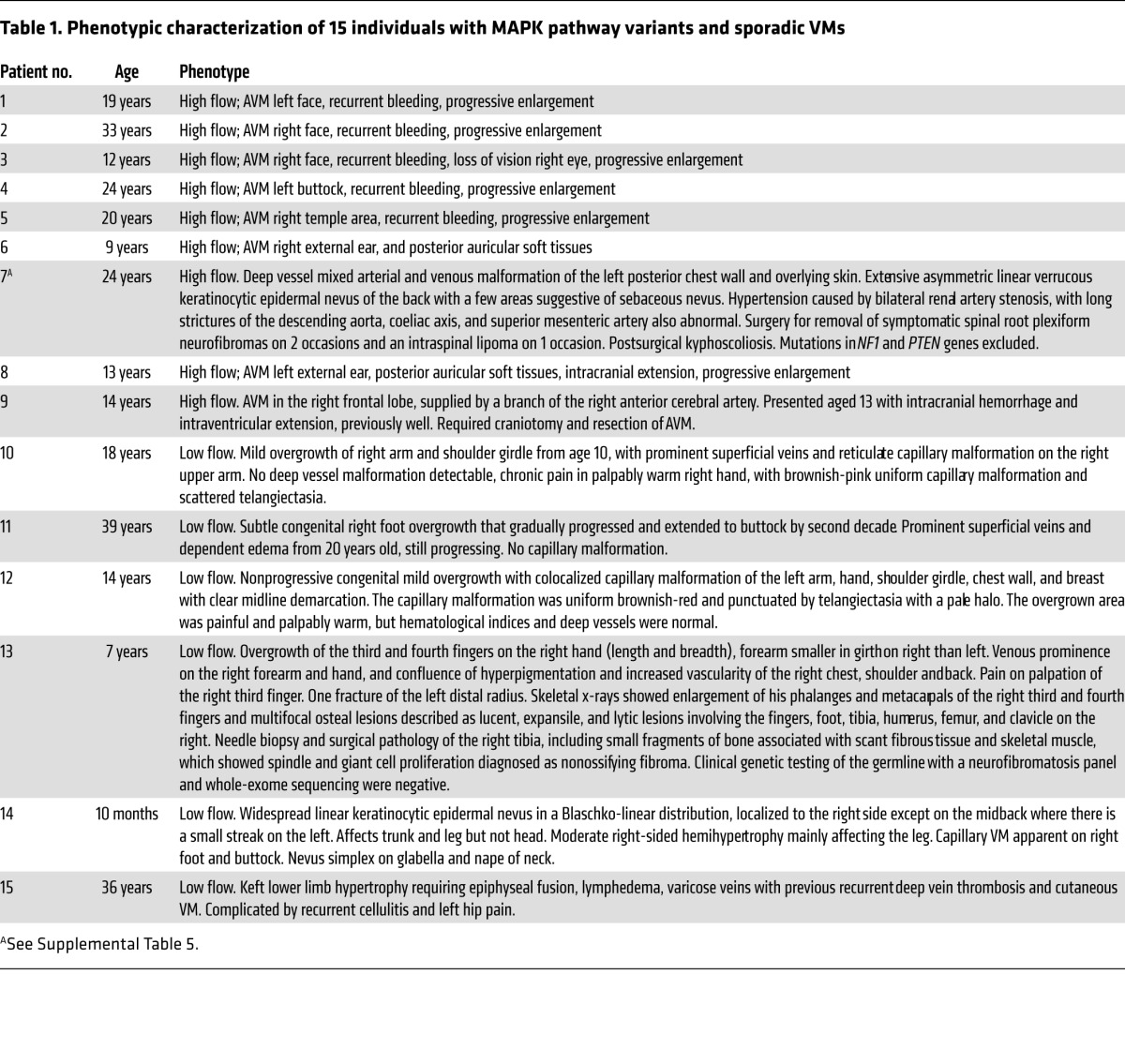
Phenotypic characterization of 15 individuals with MAPK pathway variants and sporadic VMs

**Table 2:**
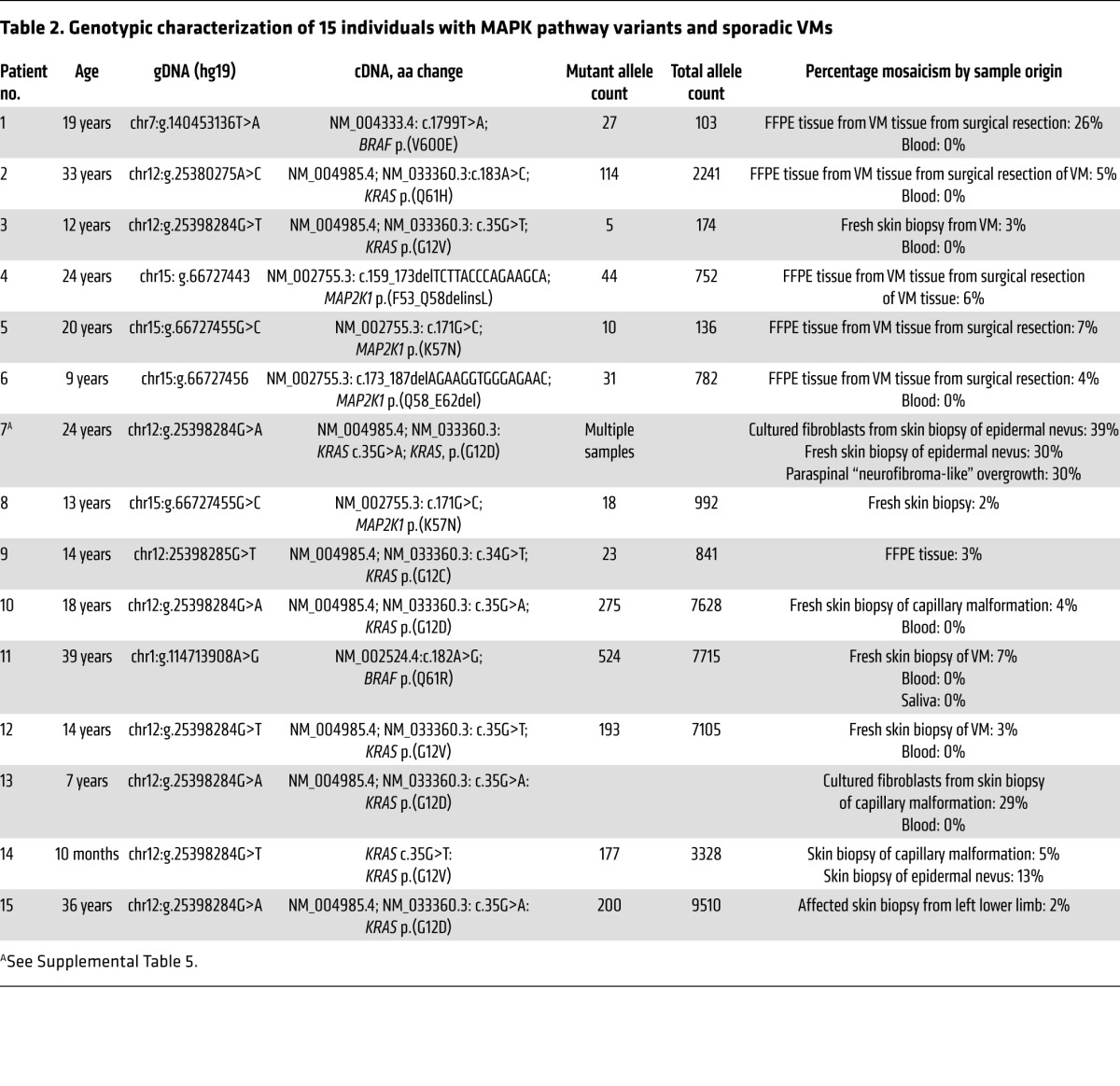
Genotypic characterization of 15 individuals with MAPK pathway variants and sporadic VMs

## Version 1 02/20/2018

In-Press Preview

## Version 2 03/12/2018

Electronic publication

## Version 3 04/02/2018

Print issue publication
