# Supplementary material for: Mosaic RAS/MAPK variants cause sporadic vascular malformations which respond to targeted therapy
Source: J Clin Invest. 2018 Mar 12;128(4):1496–508. doi: 10.1172/JCI98589 (PMC5873857; doi:10.1172/JCI98589)
Supplement: Supplemental data [file jci-128-98589-s001.pdf]

SUPPLEMENTARY MATERIAL

Figure S1 – the effects of BRAF overexpression on tail development in zebrafish larvae.
(a) Image of an example of a zebrafish larvae expressing high levels of MAPK signaling, leading to a "stunting" phenotype, as previously described(1, 2) (b) Graph of zebrafish embryos expressing BRAF WT or V600E alleles. Both WT and mutant alleles lead to a stunted phenotype that confounds analysis of the vasculature in those animals, and were excluded from further analysis.

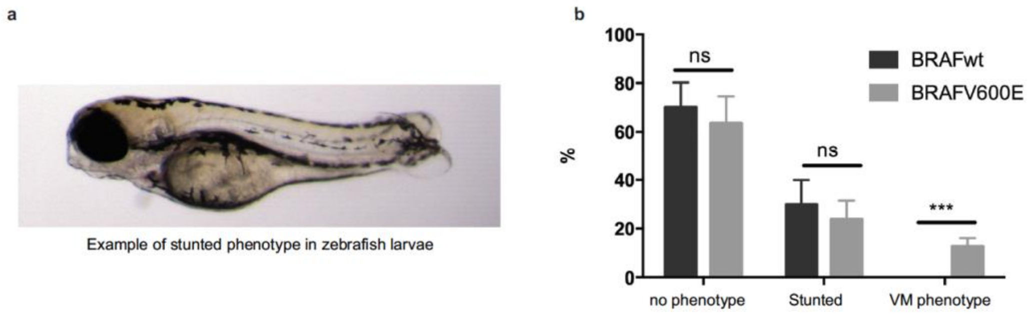

Figure S2 - ***In silico*** **modelling using STRING of networks of genes implicated in human vascular malformations gene products.**

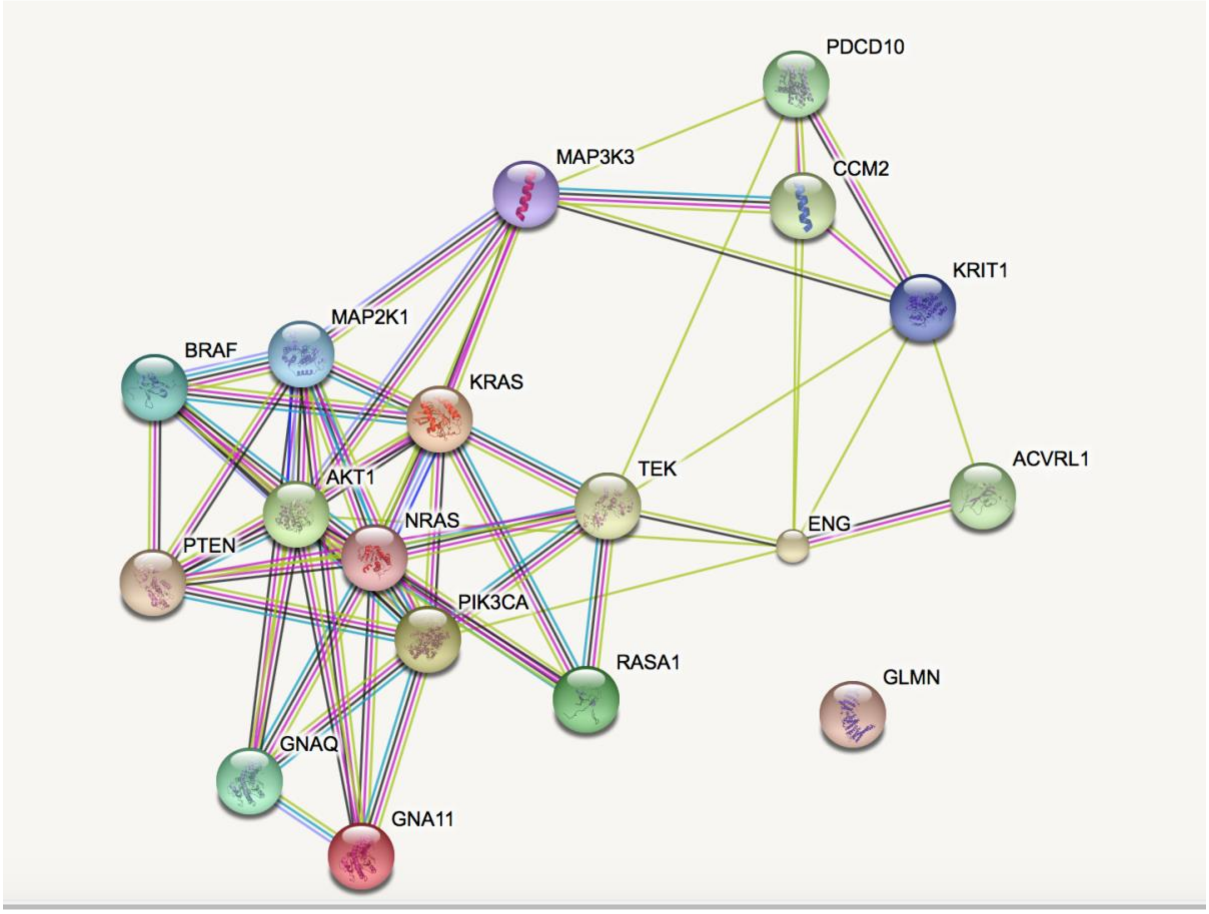

Table S1: Primers used for Sanger sequencing

Mosaic variant	Forward primer sequence (5' to 3')	Reverse primer sequence (5' to 3')
KRAS<br>c.35G>A, p.Gly12Asp	AGCGTCGATGGAGGAGTTTG	ACAGAGAGTGAACATCATGGACC
MAP2K1<br>• c.171G>C, p.(K57N)<br>• c.159_173delTCTTACCCAGAAGCA, p.(F53_Q58delinsL)<br>• c.173_187delAGAAGGTGGGAGAAC, p.(Q58_E62del)	TGGGTTGACTTCTCTGGTGA	GAGACCTTGAACACCACACC

Table S2: Gene targets covered by the custom-designed 60 gene overgrowth sequencing panel (AmpliSeq, Life Technologies).

195 amplicons ranging from 125-275 bp in size were designed to cover the codons of interest listed below, except in the case of *PIK3CA*, *PTEN* and *CCND2*, where full coverage of all coding regions is provided. Annotations are according to reference genome hg19. Where multiple transcript variants exist, the longest transcript variant is stated for the purpose of amino acid numbering.

Gene	Regions covered	Amino acids covered
AKT1	Chr14: 105246445 – 105246583	p.G16 – p.L52
	Chr14: 105241352 – 105241535	p.E151 – p.K189
AKT2	Chr19: 40762852 – 40763002	p.G16 – p.L52
AKT3	Chr1: 243858913 – 243859106	p.G16 – p.L51
ALK	Chr2: 29443490 – 29443714	p.K1173 – p.P1215
	Chr2: 29436788 – 29437003	p.S1216 – p.H1247
	Chr2: 29432582 – 29432804	p.D1249 – p.Y1278
BRAF	Chr7: 140481298 – 140481511	p.K439 – p.H477
	Chr7: 140453028 – 140453243	p.I582 – p.M620
CCND2	All coding regions	
CDK2	Chr12: 56361535 – 56361762	p.E40 – p.V64
	Chr12: 56362526 – 56362676	p.Y107 – p.L143
DEPTOR	Chr8: 121018956 – 121019175	p.L310 – p.T332
EGFR	Chr7: 55241601 – 55241801	p.L688 – p.K728
	Chr7: 55248900 – 55249123	p.E762 – p.D807
	Chr7: 55249121 – 55249200	p.D807 – p.K823
	Chr7: 55259353 – 55259582	p.G824 – p.K875
ERBB2	Chr17: 37868167 – 37868373	p.N302 – p.R340
	Chr17: 37880147 – 37880352	p.G737 – p.D769
ERBB3	Chr12: 56478786 – 56479009	p.R81 – p.A155
EZH2	Chr7: 148508619 – 148508841	p.H618 – p.E649
FGFR1	Chr8: 38285848 – 38286070	p.A121 – p.M149
	Chr8: 38282094 – 38282318	p.R281 – p.L321
FGFR2	Chr10: 123279547 – 123279766	p.R251 – p.E295
	Chr10: 123274672 – 123274890	p.P364 – p.V416
	Chr10: 123257952 – 123258121	p.D523 – p.D558
FGFR3	Chr4: 1803433 – 1803647	p.G235 – p.C275
	Chr4: 1806061 – 1806208	p.E362 – p.V413
FLT3	Chr13: 28610030 – 28610230	p.K438 – p.P472
	Chr13: 28608204 – 28608422	p.Q569 – p.F612
	Chr13: 28602195 – 28602418	p.K649 – p.S684
	Chr13: 28592526 – 28592736	p.C807 – p.N847
GNA11	Chr19: 3118790 – 3118993	p.M203 – p.I226

GNAQ	Chr9: 80409380 – 80409598	p.M203 – p.E245
GNAS	Chr20: 57484401 – 57484626	p.D839 – p.F862
HRAS	Chr11: 534102 – 534306	p.K5 – p.E37
	Chr11: 533780 – 533936	p.R41 – p.I93
IDH1	Chr2: 209113051 – 209113255	p.E84 – p.Q138
IDH2	Chr15: 90631752 – 90631976	p.F126 – p.Q178
IGF1R	Chr15: 99440011 – 99440237	p.E325 – p.G367
IGF2R	Chr6: 160485824 – 160485949	p.P1340 – p.F1371
	Chr6: 160496879 – 160497103	p.D1723 – p.M1772
	Chr6: 160517434 – 160517663	p.G2220 – p.L2280
JAK2	Chr9: 5073668 – 5073862	p.S593 – p.E621
JAK3	Chr19: 17954041 – 17954267	p.E113 – p.Q140
	Chr19: 17947870 – 17948080	p.S568 – p.D595
	Chr19: 17945623 – 17945811	p.L684 – p.K733
KDR	Chr4: 55980271 – 55980456	p.Y221 – p.K266
	Chr4: 55979507 – 55979727	p.Q268 – p.M314
	Chr4: 55972826 – 55973041	p.Q472 – p.K512
	Chr4: 55962395 – 55962621	p.G873 – p.G909
	Chr4: 55960908 – 55961127	p.T940 – p.E990
	Chr4: 55955033 – 55955251	p.Y1136 – p.Q1170
	Chr4: 55953691 – 55953917	p.G1172 – p.I1220
	Chr4: 55946189 – 55946355	p.G1284 – p.I1330
	Chr4: 55946011 – 55946232	p.I1330 – p.V1356
KIT	Chr4: 55561667 – 55561888	p.S24 – p.G93
	Chr4: 55592066 – 55592292	p.S464 – p.K513
	Chr4: 55593338 – 55593546	p.Q515 – p.Q549
	Chr4: 55593499 – 55593700	p.K550 – p.L589
	Chr4: 55594136 – 55594358	p.S628 – p.G663
	Chr4: 55595409 – 55595630	p.P665 – p.L707
	Chr4: 55597389 – 55597603	p.S715 – p.I744
	Chr4: 55599232 – 55599442	p.C788 – p.N828
	Chr4: 55602591 – 55602803	p.A829 – p.L865
KRAS	Chr12: 25398183 – 25398385	p.M1 – p.E37
	Chr12: 25380260 – 25380337	p.R41 – p.A66
	Chr12: 25378561 – 25378743	p.E98 – p.A146
LAMTOR1	Chr11: 71810184 – 71810406	p.D15 – p.L54
	Chr11: 71809744 – 71809973	p.N64 – p.Y88
LAMTOR2	Chr1: 156025008 – 156025204	p.L24 – p.M74
MAP2K1	Chr15: 66729047 – 66729265	p.L98 – p.M146
	Chr15: 66774032 – 66774239	p.V191 – p.S231
	Chr15: 66727373 – 66727563	p.T28 – p.A95

MAPKAP1	Chr9: 128246741 – 128246970	p.S357 – p.F396
	Chr9: 128201149 – 128201292	p.V482 – p.Q522
MET	Chr7: 116339530 – 116339753	p.I131 – p.F206
	Chr7: 116340170 – 116340389	p.G344 – p.R400
	Chr7: 116403120 – 116403338	p.S812 – p.K879
	Chr7: 116411806 – 116412001	p.L982 – p.V1014
	Chr7: 116417424 – 116417614	p.H1106 – p.N1131
	Chr7: 116423291 – 116423508	p.L1230 – p.T1280
MLST8	Chr16: 2256502 – 2256711	p.Q63 – p.L114
	Chr16: 2258233 – 2258463	p.L199 – p.S232
MTOR	Chr1: 11190760 – 11190931	p.A1789 – p.E1813
	Chr1: 11190565 – 11190770	p.Q1807 – p.E1871
	Chr1: 11187658 – 11187880	p.V2012 – p.Q2072
	Chr1: 11174371 – 11174529	p.V2389 – p.D2433
	Chr1: 11169263 – 11169482	p.G2484 – p.T2509
NRAS	Chr1: 115258627 – 115258821	p.M1 – p.E37
	Chr1: 115256452 – 115256669	p.D38 – p.S87
	Chr1: 115252147 – 115252254	p.T127 – p.Q150
PDGFRA	Chr4: 55140927 – 55141154	p.K552 – p.L595
	Chr4: 55144059 – 55144277	p.T632 – p.S667
	Chr4: 55151970 – 55152162	p.C814 – p.S854
PDK1	Chr2: 173427380 – 173427601	p.R138 – p.M156
	Chr2: 173450928 – 173451139	p.M336 – p.L372
	Chr2: 173457684 – 173457906	p.P380 – p.K410
PHLPP1	Chr18: 60642573 – 60642794	p.K1253 – p.S1307
PHLPP2	Chr16: 71683541 – 71683769	p.A999 – p.G1075
PIK3CA	All coding regions	
PIK3CB	Chr3: 138374192 – 138374406	p.D1026 – p.S1070
PIK3R1	Chr5: 67593218 – 67593445	p.V663 – p.R724
PIK3R2	Chr19: 18273734 – 18273817	p.K371 – p.G385
PTEN	All coding regions	
PTPN11	Chr12: 112888117 – 112888297	p.R47 – p.C104
	Chr12: 112926780 – 112926999	p.V484 – p.Q533
RHEB	Chr7: 151187964 – 151188182	p.K19 – p.N41
	Chr7: 151167603 – 151167827	p.V128 – p.Q154
RICTOR	Chr5: 38959878 – 38959945	p.I663 – p.Q683
	Chr5: 38952408 – 38952632	p.T967 – p.D1006
	Chr5: 38950550 – 38950776	p.S1058 – p.L1134
RPS6KB1	Chr17: 58011763 – 58011967	p.A261 – p.A290
	Chr17: 58013815 – 58014032	p.A348 – p.L373
RPS6KB2	Chr11: 67201650 – 67201787	p.R324 – p.L349

RPTOR	Chr17: 78882538 – 78882765	p.V802 – p.K840
	Chr17: 78899135 – 78899346	p.T937 – p.K973
	Chr17: 78933883 – 78934090	p.D1160 – p.E1201
RRAGA	Chr9: 19049675 – 19049856	p.T4 – p.Q66
RRAGB	ChrX: 55748556 – 55748778	p.V43 – p.T75
	ChrX: 55757816 – 55758046	p.E132 – p.L200
	ChrX: 55779786 – 55780009	p.A233 – p.L273
RRAGC	Chr1: 39322645 – 39322863	p.V80 – p.D116
	Chr1: 39322526 – 39322746	p.D116 – p.Q147
	Chr1: 39321410 – 39321639	p.D148 – p.A204
RRAGD	Chr6: 90077747 – 90077976	p.L352 – p.L400
SMAD4	Chr18: 48575081 – 48575265	p.H92 – p.D142
	Chr18: 48575548 – 48575763	p.D142– p.N151
	Chr18: 48581005 – 48581225	p.A152 – p.H177
	Chr18: 48584490 – 48584654	p.S223 – p.H262
	Chr18: 48586137 – 48586349	p.P303 – p.P318
	Chr18: 48591742 – 48591964	p.A319 – p.I376
	Chr18: 48593403 – 48593514	p.G384 – p.P422
	Chr18: 48603025 – 48603205	p.Q442 – p.I482
	Chr18: 48604603 – 48604826	p.S483 – p.P550
SOS1	Chr2: 39281647 – 39281866	p.E198 – p.N240
	Chr2: 39249808 – 39250023	p.E515 – p.I587
SRC	Chr20: 36031585 – 36031773	p.R472 – p.E534
STAT3	Chr17: 40474350 – 40474557	p.K631 – p.G684

Table S3: Restriction fragment length polymorphism assay reagents.
PCR products were designed to cover the codon of interest. One primer was labelled with 6-carboxyfluorescein (6FAM) for detection via Genemapper software on an AB13730 capillary sequencer. The other primer was designed to include a restriction enzyme recognition site (altered bases depicted in capitals), resulting in specific digestion of the mutant but not the wildtype allele.

Mosaic variant	Forward primer sequence (5' to 3')	Reverse primer sequence (5' to 3')	Restriction enzyme	Size of wildtype fragment (bp)	Size of mutant fragment following digestion (bp)
KRAS, c.35G>A, p.Gly12Asp	aaacttgtggtagttggagcGg	[6FAM]aagaatggtcctgcaccagta	FokI	145	111
NRAS c.182A>G, p.Gln61Arg	gacatactggatacagcCgTac	[6FAM]tggggaaatgaggttaccaca	BsiWI	206	188
KRAS c.35G>T, Gly12Val	[6FAM]aaaaggtactggtggagtatttga	tcaaggcactcttgcctacgTTa	HpaI	158	136

Table S4: Antibodies used for immunoblotting

Antibody name	Dilution	Catalogue number
MAP2K1 (MEK1) Antibody	1:1000	Cell Signalling Technology #9124
p44/p42 MAPK (Erk1/2)(137F5) Rabbit mAb	1:500	Cell Signalling Technology #4695
Phospho-p44/42 MAPK (Erk1/2)(Thr202/Tyr204)(197G2) Rabbit mAb	1:1000	Cell Signalling Technology #4377
GAPDH (14C10) Rabbit mAb	1:3000	Cell Signalling Technology #2118
Anti-Rabbit IgG (γ-chain specific)–Peroxidase antibody, Mouse monoclonal RG-96 (secondary antibody used in all immunoblotting experiments)	1:7000	Sigma *A1949*

Table S5: Results of genotyping for *PIK3CA* and *KRAS* in multiple tissue samples in patient 7. MAF = minor allele frequency; FFPE = formalin-fixed, paraffin-embedded; RFLP = fluorescent Restriction Fragment Length Polymorphism assay; NGS = next generation sequencing

**Sample**	**Genotype**	**MAF**	**Technique**	**Comment**
Intradural, extra-medullary spinal plexiform tumour (FFPE)	*PIK3CA* H1047L<br><br>*KRAS* G12D	7%<br>0%<br>0%	RFLP<br>NGS<br>NGS	Poor quality FFPE DNA. *PIK3CA* variant only present in 1 FFPE block out of 4. Low read depth in NGS (mean read depth < 50)
Neurofibroma (FFPE)	*KRAS* G12D<br>*PIK3CA* H1047L	30%<br>0%	Sanger<br>Sanger, RFLP	
Lipoma (FFPE)	*PIK3CA* H1047L	3%<br>0%	RFLP<br>Sanger	Poor quality FFPE DNA.
Epidermal nevus fresh tissue	*KRAS* G12D<br>*PIK3CA* H1047L	30%<br>0%	NGS, Sanger<br>NGS, RFLP	
Fibroblasts grown from epidermal nevus	*KRAS* G12D<br>*PIK3CA* H1047L	39%<br>0%	NGS, Sanger<br>NGS, RFLP	
Blood	*PIK3CA* H1047L<br>*KRAS* WT	0%<br>0%	RFLP, Sanger<br>Sanger	

References

1. Anastasaki C, Estep AL, Marais R, Rauen KA, and Patton EE. Kinase-activating and kinase-impaired cardio-facio-cutaneous syndrome alleles have activity during zebrafish development and are sensitive to small molecule inhibitors. *Human molecular genetics.* 2009;18(14):2543-54.
2. Anastasaki C, Rauen KA, and Patton EE. Continual low-level MEK inhibition ameliorates cardio-facio-cutaneous phenotypes in zebrafish. *Dis Model Mech.* 2012;5(4):546-52.
